# Distribution of gyrA, rrs, and eis Gene Mutations Associated With Second-Line Drug Resistance Among Multidrug-Resistant Mycobacterium tuberculosis Isolates

**DOI:** 10.7759/cureus.115987

**Published:** 2026-09-09

**Authors:** Rinku Yadav, Rosy Bala, Aparna Parmar, Narinder Kaur, Parminder Singh

**Affiliations:** 1 Microbiology, Maharishi Markandeshwar Institute of Medical Sciences and Research (MMIMSR), Maharishi Markandeshwar (Deemed to be University), Ambala, IND; 2 Microbiology, Pandit Bhagwat Dayal Sharma Post Graduate Institute of Medical Sciences (PGIMS), Rohtak, IND

**Keywords:** drug-resistant tuberculosis, fluoroquinolone resistance, genotype mtbdrsl, gyra, mdr-tb, molecular diagnostics, pre-xdr tb, rrs, second-line injectable drug resistance

## Abstract

Background: Drug-resistant tuberculosis (DR-TB) remains a major public health challenge, particularly with the increasing prevalence of resistance to second-line anti-tubercular drugs. Rapid detection of mutations associated with fluoroquinolone (FQ) and second-line injectable drug (SLID) resistance is essential for appropriate treatment selection and prevention of further drug resistance.

Objectives: To determine the genotypic patterns of second-line drug resistance among drug-resistant *Mycobacterium tuberculosis* isolates using GenoType MTBDRsl Version 2.0 (Hain Lifescience GmbH (a Bruker company), Nehren, Germany) and to characterize mutations associated with resistance to FQs and SLIDs.

Methods: A cross-sectional study was conducted on 85 drug-resistant *Mycobacterium tuberculosis* isolates. GenoType MTBDRsl Version 2.0 assay was performed to detect mutations in the *gyrA*, *gyrB*, *rrs*, and *eis* genes associated with resistance to FQs and SLIDs. Isolates were categorized according to World Health Organization (WHO) resistance definitions.

Results: A total of 85 drug-resistant *Mycobacterium tuberculosis* isolates were analyzed, including 42 (49.4%) isoniazid-resistant TB (Hr-TB), 14 (16.5%) rifampicin-resistant TB (RR-TB), and 29 (34.1%) multidrug-resistant TB* (*MDR-TB) isolates. FQ resistance was detected in 30 (35.3%) isolates, while seven (8.2%) were resistant to SLIDs. Based on final classification, 33 (38.8%) isolates were Hr-TB, nine (10.6%) Hr-TB with FQ resistance, nine (10.6%) RR-TB, five (5.9%) RR-TB with FQ resistance, 13 (15.3%) MDR-TB, and 16 (18.8%) pre-extensively drug-resistant (pre-XDR) TB. Among FQ-resistant isolates, the most common mutation was *gyrA* D94G (53.3%), followed by A90V (20.0%) and D94N/Y (16.7%). Among SLID-resistant isolates, *rrs* mutations predominated, with A1401G detected in 28.6% and G1484T in 14.3% of isolates. No isolated *eis* promoter mutations were observed.

## Introduction

The aim of the study was to determine the genotypic characterization and drug resistance profile of multidrug-resistant* *tuberculosis (MDR-TB) from clinically diagnosed pulmonary TB patients. 

*Mycobacterium tuberculosis (M. tuberculosis)* is the infectious agent responsible for TB. It is presently the second most common cause of death from infectious diseases, following coronavirus disease 2019 (COVID-19), and the 13th most common cause of death overall, according to the most recent World Health Organization (WHO) reports [1]. TB is one of the top causes of death from a single infectious agent worldwide, with an expected 10.7 million cases and 1.23 million deaths from the disease in 2024, according to the WHO Global Tuberculosis Report 2025 [2]. Global attempts to eliminate TB are still threatened by drug-resistant TB (DR-TB), especially rifampicin-resistant TB (RR-TB), MDR-TB, and extensively DR-TB (XDR-TB). Isoniazid-resistant TB (Hr-TB), RR-TB, MDR-TB, pre-XDR-TB, and XDR-TB are the five types of TB drug resistance. MDR-TB is defined as TB that is resistant to the two most potent first-line anti-TB medications, isoniazid (INH) and rifampicin (RIF). XDR-TB is defined as MDR-TB plus resistance to any fluoroquinolone (FQ) and at least one additional group A medication (linezolid and bedaquiline) [3]. Pre-XDR-TB is MDR-TB that is resistant to any FQ.

DR-TB continues to have a significant worldwide burden. Globally, an estimated 390,000 people contracted MDR/RR-TB in 2024. Four nations accounted for more than half of these cases: the Russian Federation (6.7%), China (7.1%), the Philippines (7.1%), and India (32%). India is still the country with the highest rate of TB worldwide and a significant source of DR-TB patients [2].

The India TB Report 2024 states that 11,749 pre-XDR-TB and 114 XDR-TB cases were among the 63,929 MDR/RR-TB cases diagnosed in India in 2023. Furthermore, 23,019 cases of INH mono/poly-resistant TB were documented [4]. Diagnosis, treatment, and disease control are severely hampered by the rising incidence of DR-TB. In order to start the right treatment, stop the spread of resistance, and stop its amplification, early diagnosis of drug resistance is essential [5].

DR-TB is managed in large part by FQs, especially moxifloxacin, which are essential parts of current MDR-TB therapy regimens [6]. However, the development of FQ resistance reduces the effectiveness of treatment and raises the possibility of developing pre-XDR and XDR-TB. For efficient patient care, it is crucial to quickly identify resistance to FQs and second-line injectable medications [7].

Mutations linked to resistance to first-line and second-line anti-tubercular medications can be quickly identified using commercial molecular assays like GenoType MTBDRplus Version 2.0 and GenoType MTBDRsl Version 2.0 (Hain Lifescience GmbH (a Bruker company), Nehren, Germany). GenoType MTBDRsl finds mutations in the *gyrA, gyrB, rrs,* and *eis* genes linked to resistance to FQs, aminoglycosides, and cyclic peptides, whereas GenoType MTBDRplus is used to identify resistance to RIF and INH [8]. For patients with DR-TB, these assays enable prompt initiation of suitable treatment regimens and quick molecular characterization of drug resistance.

## Materials and methods

Study design and setting

This cross-sectional collaborative study was conducted from September 2024 to February 2026 at the Culture and Drug Susceptibility Testing (C&DST) Laboratory, Pandit Bhagwat Dayal Sharma Post Graduate Institute of Medical Sciences (PGIMS), Rohtak, Haryana, India, in collaboration with the Department of Microbiology, Maharishi Markandeshwar Institute of Medical Sciences and Research (MMIMSR), Mullana, Ambala, Haryana, India. The Institutional Committee of the Maharishi Markandeshwar Institute of Medical Sciences and Research, Mullana, Ambala, issued approval IEC-3143.

Study population

Drug-resistant *Mycobacterium tuberculosis* isolates identified through routine diagnostic services and referred for second-line drug susceptibility testing during the study period were included.

Sample size

A total of 85 drug-resistant *Mycobacterium tuberculosis* isolates were included in the study.

Inclusion criteria

The study included culture-confirmed *Mycobacterium tuberculosis* isolates received during the study period that were categorized as DR-TB, including Hr-TB, RR-TB, and MDR-TB. Only those isolates with valid and interpretable GenoType MTBDRsl Version 2.0 assay results were included in the analysis.

Exclusion criteria

The study excluded non-tuberculous mycobacterial (NTM) isolates, contaminated cultures, and samples that yielded invalid, indeterminate, or uninterpretable results on the GenoType MTBDRsl Version 2.0 assay.

Culture and identification

Clinical specimens were processed according to National Tuberculosis Elimination Programme (NTEP) guidelines [9]. Isolation and identification of *Mycobacterium tuberculosis* were performed in the C&DST Laboratory, PGIMS Rohtak, using standard microbiological procedures.

GenoType MTBDRsl version 2.0 assay

A second-line probe assay (GenoType MTBDRsl Version 2.0) was performed according to the manufacturer's instructions. The assay involved: DNA extraction GenoType MTBDRplus assay kits from culture isolates; multiplex polymerase chain reaction (PCR) amplification of target gene regions; reverse hybridization of amplified products on nitrocellulose strips; and interpretation of wild-type (WT) and mutation bands.

Detection of resistance-associated mutations

The assay detects mutations in the following genes: *gyrA* and *gyrB* genes, associated with resistance to FQs (levofloxacin and moxifloxacin); *rrs* gene, associated with resistance to amikacin, kanamycin, and capreomycin; and *eis* promoter region, associated with low-level kanamycin resistance.

Interpretation of results

Resistance was interpreted according to the manufacturer's guidelines. Absence of WT bands and/or presence of mutation bands was considered indicative of resistance-associated mutations. Mutation patterns in the *gyrA, gyrB, rrs, *and* eis *genes were recorded and analyzed.

## Results

A total of 759 *Mycobacterium tuberculosis* isolates were included in the study. Of these, 674 (88.8%) isolates were susceptible to both RIF and INH. RIF monoresistance was observed in 14 (1.8%) isolates, while 29 (3.8%) isolates were resistant to both RIF and INH and were therefore classified as MDR-TB. Overall, resistance to RIF, INH, or both drugs was detected in 85 (11.2%) isolates. These findings indicate that the majority of isolates remained susceptible to first-line anti-tubercular drugs, although INH resistance was more common than RIF monoresistance.

Among the 85 drug-resistant isolates, the second-line line probe assay (GenoType MTBDRsl Ver. 2.0) identified 30 FQ-resistant isolates. Mutations in the *gyrA* gene were detected in all FQ-resistant isolates. The most prevalent mutation was D94G (MUT3C), observed in 16 (53.3%) isolates, followed by A90V (MUT1) in six (20.0%) isolates and D94N/Y (MUT3B) in five (16.7%) isolates. Less frequent mutations included D94A (MUT3A) in two (6.7%) isolates and S91P (MUT2) in one (3.3%) isolate. No isolates exhibited the D94H (MUT3D) mutation or mutations in the *gyrB* gene (N538D and E540V) (Table 1).

**Table 1:** Distribution of gyrA and gyrB gene mutation patterns associated with fluoroquinolone resistance Detected by GenoType MTBDRsl Version 2.0 (Hain Lifescience GmbH (a Bruker company), Nehren, Germany). WT: wild-type; MUT: mutation

Gene	Codon/Region	Mutation Pattern	Levofloxacin Resistance	Moxifloxacin Resistance	No. of Isolates	Percentage (%)
gyrA	A90V	WT2 absent + MUT1 present	Resistance	Low-level	6	20.0
gyrA	S91P	WT3 absent + MUT2 present	Resistance	Low-level	1	3.3
gyrA	D94A	WT3/WT4 absent + MUT3A present	Resistance	Low-level	2	6.7
gyrA	D94N/Y	WT3/WT4 absent + MUT3B present	Resistance	High-level	5	16.7
gyrA	D94G	WT3/WT4 absent + MUT3C present	Resistance	High-level	16	53.3
gyrA	D94H	WT3/WT4 absent + MUT3D present	Resistance	High-level	-	-
gyrB	N538D	WT absent + MUT1 present	Resistance	Usually Low-level	-	-
gyrB	E540V	WT absent + MUT2 present	Resistance	Usually Low-level	-	-

Analysis of FQ resistance levels revealed that the A90V, S91P, and D94A mutations were associated with low-level moxifloxacin resistance, whereas D94N/Y and D94G mutations were associated with high-level moxifloxacin resistance. The predominance of the D94G mutation highlights a substantial burden of high-level FQ resistance among the study isolates.

A total of seven isolates demonstrated resistance to second-line injectable drugs (SLIDs). Mutations in the *rrs* gene constituted the predominant resistance mechanism. The A1401G (MUT1) mutation was identified in two (28.6%) isolates, while the G1484T (MUT2) mutation was detected in one (14.3%) isolate. Additionally, three (42.9%) isolates exhibited loss of the *rrs* WT band without a corresponding mutation band, suggesting the presence of uncharacterized mutations within the 1401-1402 region. One isolate (14.3%) showed combined *rrs* and *eis* gene mutations, indicating resistance to aminoglycosides and cyclic peptide agents. No isolated *eis* promoter mutations were observed (Table 2).

**Table 2:** Distribution of rrs and eis gene mutation patterns among SLID-resistant isolates Detected by GenoType MTBDRsl Version 2.0 (Hain Lifescience GmbH (a Bruker company), Nehren, Germany). SLID: second-line injectable drug; WT: wild-type; MUT: mutation

Gene	WT Band Missing	MUT Band Present	Mutation Interpreted	Resistance Pattern	No. of Isolates (n=07)	Percentage (%)
rrs	WT1	MUT1	A1401G	AMK, KAN, CAP resistance	2	28.6
rrs	WT2	MUT2	G1484T	AMK, KAN, CAP resistance	1	14.3
rrs	WT1 absent only	No MUT band	Unknown mutation in 1401/1402 region	SLID resistance	3	42.9
eis	WT1	MUT1	C-14T	Low-level KAN resistance	-	-
eis	WT2	No MUT band	C-12T or G-10A	Low-level KAN resistance	-	-
eis	WT3	No MUT band	G-37T	Low-level KAN resistance	-	-
eis	WT4	No MUT band	A-2C	Low-level KAN resistance	-	-
rrs + eis	WT1 + WT2	MUT1 + MUT1	Combined mutations	High-level AMK/CAP + KAN resistance	1	14.3
Wild-type	None	None	No mutation detected	Susceptible pattern	-	-
Total	—	—	—	—	7	100.0

Classification of the 85 drug-resistant isolates based on first-line and second-line drug resistance patterns revealed that Hr-TB was the most common category, accounting for 33 (38.8%) isolates. An additional nine (10.6%) isolates were classified as Hr-TB with FQ resistance. RR-TB and RR-TB with FQ resistance were identified in nine (10.6%) and five (5.9%) isolates, respectively. MDR-TB was observed in 13 (15.3%) isolates, while 16 (18.8%) isolates fulfilled the criteria for pre-XDR-TB. Overall, FQ resistance was detected across multiple resistance categories, emphasizing the growing burden of second-line drug resistance and the importance of rapid molecular diagnostics for timely detection and appropriate treatment selection.

## Discussion

Early molecular detection of DR-TB is essential for prompt diagnosis, initiation of effective therapy, and improved patient outcomes. In the present study, 11.2% of *Mycobacterium tuberculosis* isolates exhibited resistance to RIF, INH, or both drugs, while 3.8% were classified as MDR-TB. These findings are comparable to previous Indian studies. Shivekar SS et al. reported 1,084 MDR-TB cases, accounting for 5.35% of the isolates studied [10], whereas Kumar et al. reported MDR-TB in 16 (19.51%) isolates [11]. Although the prevalence of MDR-TB in our study was lower, the findings highlight the persistent burden of DR-TB.

In the present study, the D94G mutation was the most common *gyrA* mutation, accounting for 53.3% (16/30) of FQ-resistant isolates, followed by A90V (20.0%), D94N/Y (16.7%), D94A (6.7%), and S91P (3.3%). These findings are comparable to the study by Singh et al. (2021), which reported D94G (45.9%) as the predominant mutation, followed by A90V (21.2%) among fluoroquinolone-resistant M. tuberculosis isolates [12]. The similar predominance of D94G in both studies highlights its major role in conferring high-level fluoroquinolone resistance. Furthermore, the comparable frequency of A90V suggests a consistent mutation pattern among FQ-resistant TB isolates in the Indian population. 

Similar observations were made by Mujuni D et al., who reported FQ resistance in 70 of 102 (68.6%) DR-TB isolates, with D94G (42.6%) and A90V (41.1%) being the most prevalent *gyrA* mutations [13]. Likewise, Polu VG et al. observed mutations in the *gyrA* gene in 77.9% (67/86) of FQ mono-resistant isolates and identified D94G (27.9%) and A90V (18.6%) as the most common mutations [14]. The similarity of mutation patterns across these studies confirms that D94G and A90V are the predominant *gyrA* mutations associated with FQ resistance in *Mycobacterium tuberculosis.*

Among the seven SLID-resistant isolates, mutations in the *rrs* gene were the predominant mechanism of resistance. The most common finding was the absence of the *rrs* WT1 band without a corresponding mutation band, observed in 42.9% (3/7) of isolates, suggesting the presence of uncharacterized mutations within the 1401-1402 region. Among the known mutations, A1401G was the most frequent, detected in 28.6% (2/7) of isolates, followed by G1484T in 14.3% (1/7) of isolates. One isolate (14.3%) exhibited combined *rrs* and *eis* mutations, indicating resistance to aminoglycosides and cyclic peptide agents. No isolated *eis* promoter mutations were detected. These findings are comparable to those reported by Ramakrishna V, who identified A1401G as the predominant mutation among SLID-resistant isolates, occurring in 92.3% (12/13) of cases, while only 7.7% (1/13) of isolates harbored an *eis* C-14T mutation [15]. Similar observations were reported by Yadav R et al., where A1401G was the most frequent mutation pattern, followed by G1484T and various *eis* promoter alterations [16]. Likewise, Gopalaswamy R et al. reported A1401G in 61.0% (36/59) of SLID-resistant isolates, G1484T in 5.1% (3/59), and loss of the *rrs* WT1 band in 22.0% (13/59) of isolates [17].

The predominance of A1401G across all studies underscores its critical role in conferring resistance to amikacin, kanamycin, and capreomycin. However, compared with previous reports, the present study demonstrated a higher proportion of isolates with absent *rrs* WT bands without corresponding mutation bands, suggesting the presence of uncommon or novel resistance-conferring mutations not covered by the GenoType MTBDRsl Ver. 2.0 assay. These findings highlight the need for further molecular characterization of such isolates to improve understanding of resistance mechanisms and strengthen diagnostic accuracy.

Limitations

The study was conducted at a limited number of tertiary care centers in Haryana, and therefore the findings may not be fully representative of the entire population of TB patients in India. Drug resistance was assessed using a line probe assay (LPA), which detects only known mutations included in the assay design. Consequently, rare or novel mutations outside the targeted regions may have been missed.

Phenotypic drug susceptibility testing and DNA sequencing were not performed for all isolates to confirm the molecular findings and characterize uncommon mutations.

## Conclusions

Most *Mycobacterium tuberculosis* isolates in the present study were susceptible to first-line anti-tubercular drugs, although significant resistance to INH, RIF, and second-line drugs was observed. INH resistance was more common than RIF monoresistance, highlighting the need for early detection of Hr-TB. Among FQ-resistant isolates, *gyrA* mutations, particularly D94G and A90V, were the predominant resistance mechanisms. Resistance to second-line injectable drugs was mainly associated with *rrs* gene mutations, especially A1401G. The detection of absent WT bands without corresponding mutation bands suggests the presence of uncommon or novel mutations. A considerable burden of MDR-TB and pre-XDR-TB was also identified. These findings demonstrate the utility of the LPA for rapid detection of drug resistance and timely initiation of appropriate treatment.
